# Immuno-imaging and -therapy in ovarian cancer and sarcoma with de novo single-chain fv-fc fusion protein targeting TEM1/CD248

**DOI:** 10.1186/2051-1426-2-S3-P219

**Published:** 2014-11-06

**Authors:** Chunsheng Li, Junying Wang, Efthymia Iliana Matthaiou, Yi Guo, Jia Hu, Ann Marie Chacko, Vladimir R Muzykantov, George Coukos

**Affiliations:** 1 Ovarian Cancer Research Center, University of Pennsylvania, Philadelphia, PA, USA; 2 Institute for Translational Medicine and Therapeutics, University of Pennsylvania, Philadelphia, PA, USA; 3 Ludwig Cancer Research Center, University of Lausanne, Lausanne, Switzerland

## 

Tumor Endothelial Marker 1 (TEM1 or CD248) has been identified as a microvascular marker of tumor angiogenesis in various human cancers. By comparing the vasculature of ovarian cancer and normal ovary, we and others [1] identified that TEM1 is overexpressed in tumor microenvironment in ovarian cancer and it could be suggested as a suitable marker for targeting tumor vasculature of cancer therapeutics. In addition, we also confirmed that TEM1 is overexpressed in cancer cells in most soft tissue sarcoma patients.

To better assist TEM1-specific theranostics, we sought to develop TEM1-specific affinity agents. From previously isolated TEM1-specific single chain antibody, scFv78 [2], we developed a panel of new multivalent variants of scFv78 to improve the thermal, serum stability and avidity. The variants includes a dimeric Fc fusion (from hu IgG1), a tetrameric CH2 fusion, a dimeric CH3 fusion and a dimeric (Fab)2 fusion. This panel was produced using mammalian cells and affinity purified from culture supernatant. The protein of scFv78-Fc exhibited higher affinity (KD = 0.14 ± 0.01nM, 15 fold higher of parental scFv78) to the target protein in live cell ELISA assay. Using the preclinical tumor vascular models we developed [3,4], pharmacokinetics and biodistribution studies in revealed that the scFv78Fc has extended serum half-life. Near infrared (NIR) immuno-imaging [5] and 124I-immunoPET imaging with scFv78-Fc demonstrated that such tracers were enriched in TEM1-positive tumors but not normal organs. In addition, scFv78Fc was conjugated to Monomethyl auristatin E (MMAE) and saporin to develop potent TEM1-targeting immunotoxins, which showed TEM1-specific toxicity in vitro and in preclinical animal models. Lastly, TEM1-targeted nanomedicine platform was developed and studied in vitro [6] and in several preclinical tumor models. scFv78-Fc targeted, shikonin-loaded PLGA nanoparticles showed higher accumulation and killing efficacy at the TEM1 positive tumors, compared to free drug and unarmed nanoparticle controls.

Taken together, our data indicate that the scFv78-Fc is suitable for development as targeted therapeutics and imaging for cancers such as ovarian cancer and sarcomas. Based upon these findings, we propose to use the immuno-imaging for detection and selection of patients suitable for immune therapies with the TEM1-targeted immunotoxin or nanoparticles; and for monitoring the efficacy of such therapies.

## References

[B1] Conejo-Garcia JR Buckanovich RJ Benencia F Vascular leukocytes contribute to tumor vascularization Blood 2005 105 679 81 10.1182/blood-2004-05-1906 15358628

[B2] Zhao A Nunez-Cruz S Li C Rapid isolation of high-affinity human antibodies against the tumor vascular marker Endosialin/TEM1, using a paired yeast-display/secretory scFv library platform J Immunol Methods 2011 363 221 32 10.1016/j.jim.2010.09.001 20837020 PMC3003766

[B3] Chacko AM Li C Nayak M Development of 124I Immuno-PET Targeting Tumor Vascular TEM1/Endosialin Journal of nuclear medicine : official publication 2014 55 Society of Nuclear Medicine 500 7 10.2967/jnumed.113.121905 PMC4089496 24525208

[B4] Li C Chacko AM Hu J Antibody-based tumor vascular theranostics targeting endosialin/TEM1 in a new mouse tumor vascular model Cancer biology & therapy 2014 15 10.4161/cbt.27825 PMC3979822 24553243

[B5] Li C Wang J Hu J Development, Optimization, and Validation of Novel anti-TEM1/CD248 Affinity Agent for Optical Imaging in Cancer Oncotarget 2014 10.18632/oncotarget.2188 PMC4196179 25051365

[B6] Matthaiou E Barar J Sandaltzopoulos R Shikonin-loaded antibody-armed nanoparticles for targeted therapy of ovarian cancer International Journal of Nanomedicine 2014 9 10.2147/IJN.S51880 PMC3998853 24790428

